# Genetic Polymorphisms Associated with Thrombotic Disease Comparison of Two Territories: Myocardial Infarction and Ischemic Stroke

**DOI:** 10.1155/2019/3745735

**Published:** 2019-10-30

**Authors:** Irma Isordia-Salas, Manuel Martínez-Marino, Paolo Alberti-Minutti, María Tania Ricardo-Moreno, Ricardo Castro-Calvo, David Santiago-Germán, José Antonio Alvarado-Moreno, Cristian Calleja-Carreño, Jesús Hernández-Juárez, Alfredo Leaños-Miranda, Abraham Majluf-Cruz

**Affiliations:** ^1^Unidad de Investigación Médica en Trombosis, Hemostasia y Aterogénesis, H.G.R. No. 1. Dr. “Carlos Mac Gregor Sánchez Navarro” Instituto Mexicano del Seguro Social, Ciudad de México, Mexico; ^2^Servicio de Urgencias, H.G.R. No. 1. Dr. “Carlos Mac Gregor Sánchez Navarro” Instituto Mexicano del Seguro Social, Gabriel Mancera No. 222, Colonia Del Valle, CP 03100 Ciudad de México, Mexico; ^3^Unidad de Investigación Médica en Medicina Reproductiva, UMAE HGO 4, Instituto Mexicano del Seguro Social, Ciudad de México, Mexico

## Abstract

**Background and Purpose:**

The thrombin-activatable fibrinolysis inhibitor (TAFI) is an important inhibitor of fibrinolysis and plays a critical role in the pathogenesis of arterial thrombosis; genetic polymorphisms of the TAFI gene affect its activity and increase the risk of thrombosis. Moreover, studies in young patients are still scarce. The aim was to examine the contribution of the Thr325Ile and Ala147Thr polymorphisms with ST acute myocardial infarction (STEMI) or idiopathic ischemic stroke (IIS) in the young Mexican population.

**Methods:**

A total of 244 patients with STEMI ≤45 years of age and 244 controls. In a second study, 250 patients with IIS ≤45 years of age were recruited, including 250 controls. In both studies, cases and controls were matched by age and sex. The polymorphisms were determined in all participants by PCR-RFLP.

**Results:**

There was significant difference in the Thr325Ile genotype distribution (*P* = 0.001) and allele frequency (*P* = 0.001) between STEMI and control groups, but no difference in the Ala147Thr genotype distribution (*P* = 0.24) and allele frequency (*P* = 0.46), neither in the Thr325Ile genotype distribution (*P* = 0.25) nor in the Ala147Thr genotype distribution (*P* = 0.46) or their allele frequencies; there was significant difference between IIS and the control group. There were independent factors for STEMI: the Ile allele (*P* = 0.01), type 2 diabetes mellitus (*P* = 0.001), hypertension (*P* = 0.001), smoking (*P* = 0.001), dyslipidemia (*P* = 0.001), and family history of atherothrombotic disease (*P* = 0.001). The independent factors for IIS were hypertension (*P* = 0.001), smoking (*P* < 0.01), and family history of atherothrombotic disease (*P* < 0.01).

**Conclusions:**

The Thr325Ile polymorphism, but no Ala147Thr polymorphism, represents an independent risk factor for STEMI in the young Mexican population.

## 1. Introduction

Myocardial infarction (MI) and ischemic stroke (IS) are leading causes of death and disability worldwide [1]. Between 5 and 10% of individuals who suffer MI or IS are 45 years of age or less, and in more than half of the cases, the etiology is not identified [2]; it represents a very important healthcare issue in our country [3–5]. Both are multifactorial diseases influenced by genetic and environmental factors and share a common pathophysiological mechanism.

Downregulation of a fibrinolytic system contributes to arterial thrombosis (AT) in both territories. Thrombin-activatable fibrinolysis inhibitor (TAFI), as an important antifibrinolytic factor, plays a critical role in a fibrinolytic system [4, 5].

TAFI forms the molecular link between the coagulation and fibrinolytic systems [6]. The TAFI is a procarboxypeptidase that attenuates fibrinolysis, decreasing the affinity of plasmin to its substrate [7]. The Thr325IIe polymorphism (rs1926447) in the gene encoding TAFI, located on chromosome 13q14.11, results in the substitution of threonine (Thr) for isoleucine (Ile) at position 325, conferring a longer half-life and an increment up to 50% in their activity [8, 9].

Also, the Ala147Thr polymorphism (rs3742264) in the *TAFI* gene has been associated with an increase of the protein levels, which explains more than 60% of the plasmatic variability [10].

TAFIa is a potent inhibitor of fibrinolysis by removing carboxy terminal-terminal lysine residues from partially degraded fibrin and by decreasing plasminogen binding on its surface [11]. In some studies, high TAFI levels were found to be protective against MI [12]. However, in other studies, TAFI levels were associated with increased risk of MI [13, 14] or IS [15], reflecting contradictory results.

Genetic components in the development of AT are much more important in young than in older patients. Therefore, the aim of our study was to investigate the association of variations of the *TAFI* gene on the risk of ST elevation MI (STEMI) or idiopathic ischemic stroke (IIS) in young patients with the first event.

## 2. Materials and Methods

### 2.1. Study Groups

In a first case-control study consecutive patients <45 years old, with STEMI were included. Diagnosis of STEMI was based on an electrocardiogram, clinical data and laboratory in accordance with the European Society of Cardiology guidelines. They were admitted to the Intensive Coronary Care Unit of the Cardiology Hospital, Centro Médico Nacional Siglo XXI, in Mexico City, between June 2012 and June 2017. Subjects without personal history of STEMI, age- gender matched, were included in the control group.

In a second independent case-control study, consecutive patients <45 years old, with IIS, were enrolled between June 2012 and June 2017. Diagnosis of IIS was defined as an acute neurologic deficit lasting longer than 24 h with confirmation by radiographic imaging (CT or MRI). Also, cardiac/transcardiac and carotid or vertebral artery sources of emboli as well as cervical arterial dissection were always excluded by means of transesophageal and transthoracic echocardiogram, magnetic resonance angiography, and Doppler ultrasound. Individuals with ≥1 thrombophilia markers were excluded. The control group was conformed by healthy volunteers and age-gender matched. All control subjects were free of a personal or family history of thromboembolic or hemorrhagic disease.

Hypertension was defined if a subject fulfilled the European Society of Cardiology criteria or they were already being treated with antihypertensive drugs. A family history of atherothrombotic disease (ATD) was defined as coronary artery disease (CAD) or IS or sudden death in a first-degree male relative younger than 55 years of age or a female relative younger than 65 years of age. Smokers were considered if they were currently smoking or had ceased within the last 12 months. Dyslipidemia was considered if they had *cholesterol* > 200 *mg*/*dl* or if they were being already treated. Individuals were considered with diabetes if they have *fasting* *glycemia* ≥ 126 *mg*/*dl* or if they were already taking antidiabetic drugs. Ethnic background of all participants of the study was Mexican-Mestizo.

We followed the methods of Isordia-Salas et al. [16].

### 2.2. Genetic Analysis

#### 2.2.1. DNA Extraction and Genotyping of Thr147Ala and Thr325Ile Polymorphisms

The primers were Ala147Thr forward (5-TTGAAACTTCCACATGCAGC-3′) and reverse (5-ATC-TTGGGCACCATTTTGAG-3′) and Thr235Ile forward (5-CACAAAGA-AAAACAGATCACACAG-3) and reverse (5-AAAGCCACC-CAATTGTGATT-3). The protocol consisted of 30 cycles of 94°C for 30 s, 60°C for 30 s, and 72°C for 1 min. The PCR product size was 456 bp, and the G (Ala) allele was digested by *BbvI* into 28 + 124 + 304 *bp*, whereas the A (Thr) allele was digested into 28 + 428 *bp*. The PCR product was 363 bp, and the C (Thr) allele was digested by *SpeI* into 118 + 245 bp whereas the T (Ile) allele was not digested at all by *SpeI*. Amplified PCR products were digested with each enzyme, resolved on 2% agarose.

### 2.3. Ethics

The study protocol was reviewed and approved by the Human Ethical Committee and Medical Research Council of the Instituto Mexicano del Seguro Social and conforms to the ethical guidelines of the 1975 Declaration of Helsinki modified in 2013. Informed written consent was obtained from all subjects before enrollment.

### 2.4. Statistical Methods

Continuous variables were expressed as *mean* ± *standard* *deviation*, and the significance of difference was determined by Student's *t*-test. Difference between categorical variables was determined with the chi-square test. The allele frequency of each polymorphism was evaluated in each group by a chi-square test according to the Hardy-Weinberg equilibrium. Adjusted OR was calculated by multivariate logistic regression analyses for Thr147Ala, Thr325Ile polymorphisms, and traditional cardiovascular risk factors (RF). A *P* value < 0.05 was considered as a statistically significant. The statistical analyses were performed using SPSS (Statistical Package for the Social Sciences) statistical software package (version 20: SPSS Inc., Chicago, IL, USA).

## 3. Results

### 3.1. Demographic Data and Conventional RF for STEMI

Baseline characteristics of 244 patients and 244 controls are shown in Table 1. The prevalence of traditional RF was high among the patient group; those associated with MI were diabetes mellitus (30.7% vs. 16.5%; OR 2.26, 95% CI 1.43-3.58, *P* = 0.001), hypertension (39.4% vs. 17.2%; OR 3.12, 95% CI 2.01-4.86, *P* = 0.001), smoking (75.4% vs. 31.0%; OR 6.78, 95% CI 4.47-10.30, *P* = 0.001), dyslipidemia (55.3% vs. 37.2%; OR 2.12, 95% CI 1.45-3.10, *P* = 0.001), and family history of ATD (35.2% vs. 14.7%; OR 3.14, 95% CI 1.98-5.01, *P* = 0.001).

The genotype and allele distribution of Thr325Ile polymorphism for MI patients and controls are shown in Table 2. There was a significant difference in the genotype distribution (*P* = 0.01) and in the allele frequency (*P* = 0.01) between both groups. For patients and controls, frequencies for the Thr allele were 77.0% and 83.6%, respectively. Univariate analysis identified higher risk for MI in those carrying the Ile allele (i.e., those with the genotypes Thr/Ile and Ile/Ile) compared to those with the Thr/Thr genotype with an OR 1.62 (CI 95% 1.12-2.36, *P* = 0.01).

There was not a significant difference in the genotype distribution and in the allele frequency of Ala147Thr between MI patients and controls (Table 3).

### 3.2. Demographic Data and Conventional RF for IIS

Baseline characteristics of 250 patients and 250 controls are shown in Table 1. Patients with IIS had a higher prevalence of conventional RF for ATD. Hypertension was higher in the group of patients (16.8% vs. 7.2%; OR 2.6, 95% CI 1.45-4.66, *P* = 0.001), current smoking (29.2% vs. 17.2%; OR 2.06, 95% CI 1.34-3.15, *P* = 0.001), and family history of ATD (26.0% vs. 14.0%; OR 2.15, 95% CI 1.36-3.4, *P* = 0.001).

Table 4 shows the genotype and allele distribution of Thr325Ile polymorphism for IIS patients and controls without significant difference between groups.

Similar results are shown in Table 5 in respect of genotype and allele distribution of Ala147Thr for IIS patients and controls.

## 4. Discussion

AT has a highly heterogeneous clinical and etiological spectrum. The specific combination between genetic and environmental RF in different stages of life probably determines the development and the onset age of thrombotic diseases such as MI or IS [17].

Pathophysiologically, MI occurs due to artery occlusion over a ruptured coronary plaque but the mechanism in IS has diverse etiologies [18] mainly in young patients [2]. Increased levels of fibrinolytic proteins could contribute to AT development at young age. It has been demonstrated that TAFI plasma concentration is highly regulated by genetic polymorphisms and increased levels of this protein has been associated with IS or CAD [8].

To our knowledge, this is the first study involving Mexican population that assess the participation of polymorphisms in genes of the fibrinolytic system in young patients with MI or IS. In the present study, we found an association between the Thr325Ile polymorphism and STEMI patients (OR 1.62, 95% CI 1.12-2.36, *P* = 0.01).

Similar results were found by Kamal et al. in Egyptian population. They identified in a group of patients with MI that genotypes of the high-risk allele (Thr/Ile (CT) and Ile/Ile (TT)) were significantly more frequent in patients compared with the control group (54.4% and 32.6% vs. 51.8% and 5.6%, respectively) and were also associated with an increased risk of MI (OR 4.95, 95% CI 1.80-13.63, *P* < 0.001) [19]. Also, González et al. reported in Spanish population a higher frequency of the Ile/Ile genotype in patients with MI compared with controls and it was associated to a higher expression of TAFI [20].

Moreover, a previous meta-analysis by Shi et al. demonstrated an increased risk of CAD in Ile325 allele carriers only under a recessive model. But they could not support the hypothesis that Ala147Thr and Thr325Ile variants had any influence on the susceptibility of CAD [21].

However, Morange et al. in a large multicentric case-control study demonstrated that Thr325Ile polymorphism of the *TAFI* gene was not associated with an increased risk of MI [22].

TAFI is frequently implicated in AT considering it as a marker of fibrinolysis and inflammation [23]. Some authors described TAFI as a part of the atherosclerosis modulation process [24]. This is based on the fact that high levels of TAFIa have been related to changes in the flow of coronary vessels [25], as to the development of premature atherosclerosis, inflammation, and platelet aggregation, due to its identification in a human atherosclerotic plaque [26].

In our study, a negative association was found between *TAFI* Ala147Thr polymorphism and both types of infarction (myocardial and stroke) in the same group of patients. In meta-analysis, Shi et al. found no association between Ala147Thr and CAD under allele, dominant, and recessive genetic models [21]. In contrast, Morange et al. published in French population that Thr147 allele was more frequent in cases than in controls (*P* = 0.02), but not in Irish people (*P* = 0.19) [27].

Also, Juhan-Vague et al. failed to found a positive association between Ala147Thr and MI [12].

On the other hand, near 20-30% of the risk in IS is not explained by these predisposing conditions in young individuals and it has been postulated that a genetic component is more relevant in this group of patients [2, 28]. Putaala had described the strength of the association of early-onset vascular RF and cardiovascular outcomes. Although these factors are less frequent in young people, this may represent a target group for extensive studies [29].

The increase in plasma concentration of TAFI has been associated with a higher risk and severity of IS [29, 30]. Although plasma levels of TAFI are determined at least 25% by several polymorphisms located in the *CPB2* gene [31], the effect of the Thr325IIe and Ala147Thr polymorphisms in the increase in cardiovascular risk is inconclusive [21].

In our study, the Ala147Thr and Thr325IIe polymorphisms of the TAFI gene were not associated with the presence of IIS in young people. Negative results were also reported by Ladenvall et al. in subjects <70 years of age with IS [15] and by Akatsu et al. in 253 patients with an average age of 80 years [32].

In contrast, Kozian et al. in a cohort of >3,300 individuals showed an association between the homozygous Ile genotypes of the TAFI gene with a higher incidence of premature IS, but not for the Ala147Thr polymorphism [33].

The inconsistency of the results suggests that patients with IS have a complex vascular genotype and ethnic background as well as a wide interaction network with environmental RF [34].

Although TAFI levels are associated with the acute IS phase, these levels are persistently high after IS, raising a question about the real determinants of its concentration because if genetic factor is not associated, there should be another link factor between its activation and function different than that in MI [30].

Potential causes of IS are more heterogeneous than for MI. In the acute phase, patients with IS must be treated within the first hours after the onset, but in MI reperfusion, therapy by percutaneous intervention or thrombolytic therapy is wider in time.

In several models, the administration of TAFI inhibitors along with the fibrinolytic agent leads to a marked improvement of clot lysis, underscoring the potential of TAFI inhibitors as adjuvants for intravenous thrombolysis. Clinical studies in patients with thrombotic disease support the idea that increased levels of TAFI and/or the enhancement of TAFI activation may represent a new RF for venous and AT. Pharmacological inhibition of the TAFI pathway may constitute a novel strategy to prevent thrombosis or to increase the efficacy of thrombolytic therapy [28].

We had previously demonstrated that 4G/5G polymorphism in the plasminogen activator inhibitor-1 gene [35], the Glu298Asp polymorphism in the endothelial nitric oxide synthase gene [36], the platelet glycoprotein GPIIIa PlA2 polymorphism [37], the D allele from the I/D polymorphism of the angiotensin-converting enzyme gene, and the M235T polymorphism of angiotensinogen gene represent independent RF for MI [38]. In contrast, the C677T polymorphism in the methylenetetrahydrofolate reductase gene [39] and the T174M in the angiotensinogen gene [37] was not associated with CAD in this group of young individuals.

Furthermore, we have previously demonstrated that Glu298Asp in the endothelial nitric oxide synthase gene [40], the C677T polymorphism in the methylenetetrahydrofolate reductase gene [41], and the M235T and T174M in the angiotensinogen gene were associated with IS. In contrast, the platelet glycoprotein IIIA PlA2 [42], 4G/5G in the plasminogen activator inhibitor I-1 [43], and the D allele from the I/D polymorphism of the angiotensin-converting enzyme gene represent independent RF in the same group of patients.

This study has multiple limitations, such as the small sample size, the lack of TAFI level measurement, and its correlation to genetic data. However, our study has several strengths: (1) in both studies, the patients with MI and IS and controls have a similar ethnic background and were matched by age and gender; (2) in the study, young patients were included to minimize the effect of long-term environmental influences on disease etiology; and (3) laboratory genotyping was performed in a blinding manner, minimizing measurement bias.

## 5. Conclusion

In conclusion, Thr325Ile polymorphism in the *TAFI* gene was associated with an increased risk for STEMI but not for IIS in young individuals. Also, modifiable RF such as smoking, hypertension, familial history of ATD, and dyslipidemia were independently associated with MI in this study. Therefore, we hypothesized that in young patients with MI, there is a hypofibrinolytic state, increased platelet aggregation, and the presence of endothelial dysfunction, which could contribute to the atherothrombotic process and formation of premature unstable atherosclerotic plaques. In contrast, in IS, endothelial dysfunction has a major influence and could be related to hypertension and decreased vasodilation. Finally, we propose different pathways in both arterial territories based on results in our population. Additional work is needed to determine the prognostic role of genetic factors associated with an increased risk of acute thrombotic disease in young individuals, especially in those with successful control of modifiable RF.

## Figures and Tables

**Table 1:** Demographic and clinical characteristics in both study groups and controls.

Variable	Myocardial infarction group				Idiopathic ischemic stroke group			
	Cases (*n* = 244)	Controls (*n* = 244)	OR (95% CI)	*P* value	Cases (*n* = 250)	Controls (*n* = 250)	OR (95% CI)	*P* value
Age (years)	41.0 ± 4.7	39.2 ± 5.0	—	NS	35.3 ± 6.7	34.8 ± 4.2	—	NS
Sex, *n* (%)								
Man	193 (78.9)	194 (79.3)	—	NS	102 (40.8)	103 (41.2)	—	NS
Woman	51 (21.1)	50 (20.7)			148 (59.2)	147 (58.8)		
BMI (kg/m^2^)	30.2 ± 3.2	28.9 ± 4.1	—	NS	30.9 ± 2.6	29.3 ± 1.7	—	NS
Diabetes mellitus, *n* (%)	75 (30.7)	40 (16.5)	2.26 (1.43-3.58)	0.001^b^	23 (9.2)	19 (7.6)	—	NS
Hypertension, *n* (%)	96 (39.4)	42 (17.2)	3.12 (2.01-4.86)	0.001^b^	42 (16.8)	18 (7.2)	2.6 (1.45-4.66)	0.001^b^
Smoking, *n* (%)	184 (75.4)	76 (31.0)	6.78 (4.47-10.30)	0.001^b^	75 (29.2)	43 (17.2)	2.06 (1.34-3.15)	0.001^b^
Dyslipidemia, *n* (%)	135 (55.3)	90 (37.2)	2.12 (1.45-3.10)	0.001^b^	108 (43.2)	104 (44.6)	—	NS
Family history of ATD, *n* (%)	86 (35.2)	36 (14.7)	3.14 (1.98-5.01)	0.001^b^	65 (26.0)	35 (14.0)	2.15 (1.36-3.4)	0.001^b^

BMI: body mass index; ATD: atherothrombotic disease; STEMI: ST elevation myocardial infarction; CI: confidence interval; NS: not significative.

**Table 2:** Polymorphism of Thr325Ile genotype and allelic frequency in patients with myocardial infarction and controls.

	Cases (*n* = 244)	Controls (*n* = 244)	*P* value
Genotype			
Thr/Thr, *n* (%)	144 (59.0%)	171 (70.1%)	0.01^∗^
Thr/Ile, *n* (%)	88 (36.1%)	66 (27%)	
Ile/Ile, *n* (%)	12 (4.9%)	7 (2.9%)	
Ile/Ile + Thr/Ile vs. Thr/Thr	12 + 88 vs. 144	7 + 66 vs. 171	0.01^∗^
Allelic frequency			
Thr, *n* (%)	376 (77.0%)	408 (83.6%)	0.01^∗^
Ile, *n* (%)	112 (23.0%)	80 (16.4%)	

**Table 3:** Polymorphism of Ala147Thr genotype and allelic frequency in patients with myocardial infarction and controls.

	Cases (*n* = 244)	Controls (*n* = 244)	*P* value
Genotype			
Ala/Ala, *n* (%)	112 (45.9%)	120 (49.2%)	0.24
Thr/Ala, *n* (%)	103 (42.2%)	104 (42.6%)	
Thr/Thr, *n* (%)	29 (11.9%)	20 (8.2%)	
Thr/Thr + Thr/Ala vs. Ala/Ala	29 + 103 vs. 112	20 + 104 vs. 120	0.24
Allelic frequency			0.46
Ala, *n* (%)	327 (67.0%)	344 (70.5%)	
Thr, *n* (%)	161 (33.0%)	144 (29.5%)	

**Table 4:** Polymorphism of Thr325Ile genotype and allelic frequency in patients with ischemic stroke and controls.

	Cases (*n* = 250)	Controls (*n* = 250)	*P* value
Genotype			
Thr/Thr, *n* (%)	163 (65.2%)	175 (70.0%)	0.20
Thr/Ile, *n* (%)	75 (30.0%)	67 (26.8%)	
Ile/Ile, *n* (%)	12 (4.8%)	8 (3.2%)	
Ile/Ile + Thr/Ile vs. Thr/Thr	12 + 75 vs. 163	7 + 67 vs. 175	0.25
Allelic frequency			
Thr, *n* (%)	401 (77.2%)	417 (83.4%)	0.19
Ile, *n* (%)	99 (22.8%)	83 (16.6%)	

**Table 5:** Polymorphism of Ala147Thr genotype and allelic frequency in patients with ischemic stroke and controls.

	Cases (*n* = 250)	Controls (*n* = 250)	*P* value
Genotype			
Ala/Ala, *n* (%)	132 (52.8%)	118 (47.2%)	0.14
Thr/Ala, *n* (%)	101 (40.4%)	108 (43.2%)	
Thr/Thr, *n* (%)	17 (6.8%)	24 (9.6%)	
Thr/Thr + Thr/Ala vs. Ala/Ala	17 + 101 vs. 132	24 + 108 vs. 18	0.21
Allelic frequency			0.14
Ala, *n* (%)	365 (73.0%)	344 (68.8%)	
Thr, *n* (%)	135 (27.0%)	156 (31.2%)	

## Data Availability

The data used to support the findings of this study are included within the article.
